# Tannate of Mercury

**Published:** 1900-05-26

**Authors:** 


### TANNATE OF MERCURY.

Mr. Alfred Cooper, in opening a recent discussion at the West London Medico-Chirurgical Society on the treatment of syphilis, urged that in the treatment of the indurated sore, mercury should be used as soon as the diagnosis is made. He said that he places no reliance whatever on excising the indurated sore or in attempting to destroy it by any cauterant, his belief being that the induration is a local manifestation of a constitutional infection. Except, then, in cases in which the sore runs on to phagedæna, in which case the patient should be put into a hot bath until the ulcer assumes a healthy character, mercury should be given, and the sore should be dressed either with black wash or equal parts of mercurial ointment and vaseline. Of the various modes of administering mercury the routine in this country during the early stages of the disease is to administer the drug by the mouth, usually in the shape of pills or tabloids. The common form is the blue pill, three to five grains, with a quarter to half a grain of opium, twice or three times daily; or one grain of mercury and chalk with one grain of Dover's powder three or four times daily. But a preparation which Mr. Cooper has found to possess great advantages over other drugs is the tannate of mercury. This preparation is not acted upon by the gastric secretions, but passes into the duodenum unchanged, and is subsequently reacted upon by the alkaline secretions of the small intestines, and is thereby reduced to extremely fine microscopic globules of metallic mercury, so small as to allow of direct absorption by the villi of the small intestine. It does not derange the stomach to such a degree as the other mercurial preparations, and in a large majority of cases exercises no irritating influence upon the intestinal walls; it is quickly absorbed and quickly eliminated; it does not produce stomatitis to such a degree as other mercurial preparations; and since it has no accumulative effect it can be abandoned directly the gums show the least signs of soreness. It thus possesses great advantages over the substances more commonly in vogue. It may be given in doses of one or two grains three times a day in pill, and it is only exceptionally necessary to combine it with opium.

